# Interventions for healthcare providers to improve treatment and prevention of female genital mutilation: a systematic review

**DOI:** 10.1186/s12913-016-1674-1

**Published:** 2016-08-19

**Authors:** Julie Balfour, Jasmine Abdulcadir, Lale Say, Michelle J. Hindin

**Affiliations:** 1 Department of Reproductive Health and Research, World Health Organization, 20, Avenue Appia, 1211 Geneva, Switzerland; 2 Department of Obstetrics and Gynaecology, Geneva University Hospitals, Faculty of Medicine, University of Geneva, 30 Bld de la Cluse, 1211 Geneva, Switzerland

**Keywords:** Female genital mutilation, Female genital cutting, Female genital mutilation/cutting, Caregivers, Prevention, Healthcare professionals

## Abstract

**Background:**

Studies on healthcare providers’ awareness, knowledge and attitudes regarding female genital mutilation (FGM) have shown a lack of awareness of the prevalence, diagnosis, and management of FGM. Our objective was to systematically review the literature on interventions improving healthcare providers’ capacities of prevention and treatment of FGM.

**Methods:**

Systematic review of the published and grey literature on interventions aimed at improving healthcare providers’ capacities of prevention and treatment of FGM (1995–2015). Outcomes observed were knowledge and attitudes about FGM, medicalization, and prevention.

**Results:**

Only two studies met our inclusion criteria. They reported on educational interventions aimed at increasing caregivers’ knowledge on FGM. One was conducted with 59 providers, in Mali; the other one with 11 certified nurse-midwives in the United States. The studies report basic statistics regarding the improvement of healthcare professionals’ knowledge, attitude on FGM and medicalization of the practice. Neither conducted multivariable analysis nor evaluated the training effects on the quality of the care offered, the clinical outcomes and the satisfaction of women attended, and prevention.

**Conclusion:**

As health care providers are essential in prevention and treatment of FGM, developing effective interventions is crucial.

**Electronic supplementary material:**

The online version of this article (doi:10.1186/s12913-016-1674-1) contains supplementary material, which is available to authorized users.

## Background

According to the World Health Organization (WHO), female genital mutilation (FGM) involves the partial or total removal of the external female genitalia. It is a violation of human rights, has no health benefits and can be responsible for uro-gynecological, obstetric and psychosexual consequences. FGM is prevalent among some ethnic groups in Africa, Asia the Middle East, South America as well as in high income countries because of migration [1]. Medicalization refers to FGM practiced by any cadre of healthcare provider in a clinic, at home or elsewhere. It is illegal in many countries and has been condemned by the WHO, other United Nations agencies, the International Federation of Gynecology and Obstetrics and by other organizations and country governments [2].

The Green Top Guidelines of the Royal College of Obstetricians and Gynaecologists on FGM state that all clinicians should be aware of the complications of FGM and gynecologists, obstetricians and midwives should receive mandatory training on FGM and its management [3]. Some interventions indicated as possible strategies to improve the health workers’ interactions with and care of women and girls living with FGM include education to decrease medicalization, improve communication, screening, diagnosis and treatment of FGM complications [2]. Several high-income countries have recognized the need of a specific expertise in the care of women with FGM, such as defibulation during pregnancy or in labour in case of FGM type III. Specialised clinics, clinical recommendations and training tools and courses, have been implemented [4–6]. However, in spite of available learning resources, studies on caregivers’ and medical students’ awareness, knowledge and attitudes regarding FGM showed a lack of awareness of the prevalence, diagnosis, and management of FGM and difficulties in correctly classifying FGM according to the WHO classification [7]. FGM is not always included in pre- or post-grade curricula of nurses, midwives and physicians. Interventions that could improve healthcare of women with FGM and prevention of the practice have been investigated only rarely [7]. These women have specific health care needs and health care providers are essential in ensuring proper screening, diagnosis, care, counselling and prevention [8, 9].

The aim of our paper is to systematically review the available published and grey literature on the existing interventions intended at improving healthcare providers’ capacities of prevention and treatment of FGM. The results will be useful to plan future training interventions for healthcare professionals who are interacting with women and girls living with or at risk of FGM in order to improve healthcare providers’ knowledge and attitudes on FGM. The results can be used in settings with a high prevalence of FGM, and in low prevalence settings with migrant women and girls living with FGM.

## Methods

The present systematic review was conducted following the PRISMA (Preferred Reporting Items for Systematic reviews and meta-Analyses) guidelines [10]. The available published and grey literature on interventions aimed at improving healthcare providers’ capacities of prevention and treatment of FGM were identified using a predetermined WHO protocol (available on request). The systematic search included ten online databases (African Index Medicus; African Journals Online, Cochrane Library; Popline, PsychINFO/Ebscohost; Pubmed/Medline; Scopus; Web of Science; WHOLIS and Wiley Online Library) and considered publications from January 1^st^, 1995 to August 1^st^, 2015. Additional records were also retrieved through hand searching, by browsing webpages related to FGM (e.g., Population Council, TOSTAN, and IntraHealth) and through the reference lists of published systematic reviews on FGM.

The keywords used in the search were “female genital mutilation”, “female genital cutting”, “female circumcision”, “infibulation”, “health personnel”, “healthcare providers”, “nurses”, “midwives”, “doctors”, “community health workers”, “education”, “training”, “guidelines”, “knowledge”, “attitudes”, “intervention”. The terms were used in various combinations.

All studies that reported on interventions, health workers and FGM, with no language restrictions were included. JB retrieved and screened with MJH the studies for relevance to the research question. JB and JA extracted data from the included studies.

## Results

**Fig. 1:**
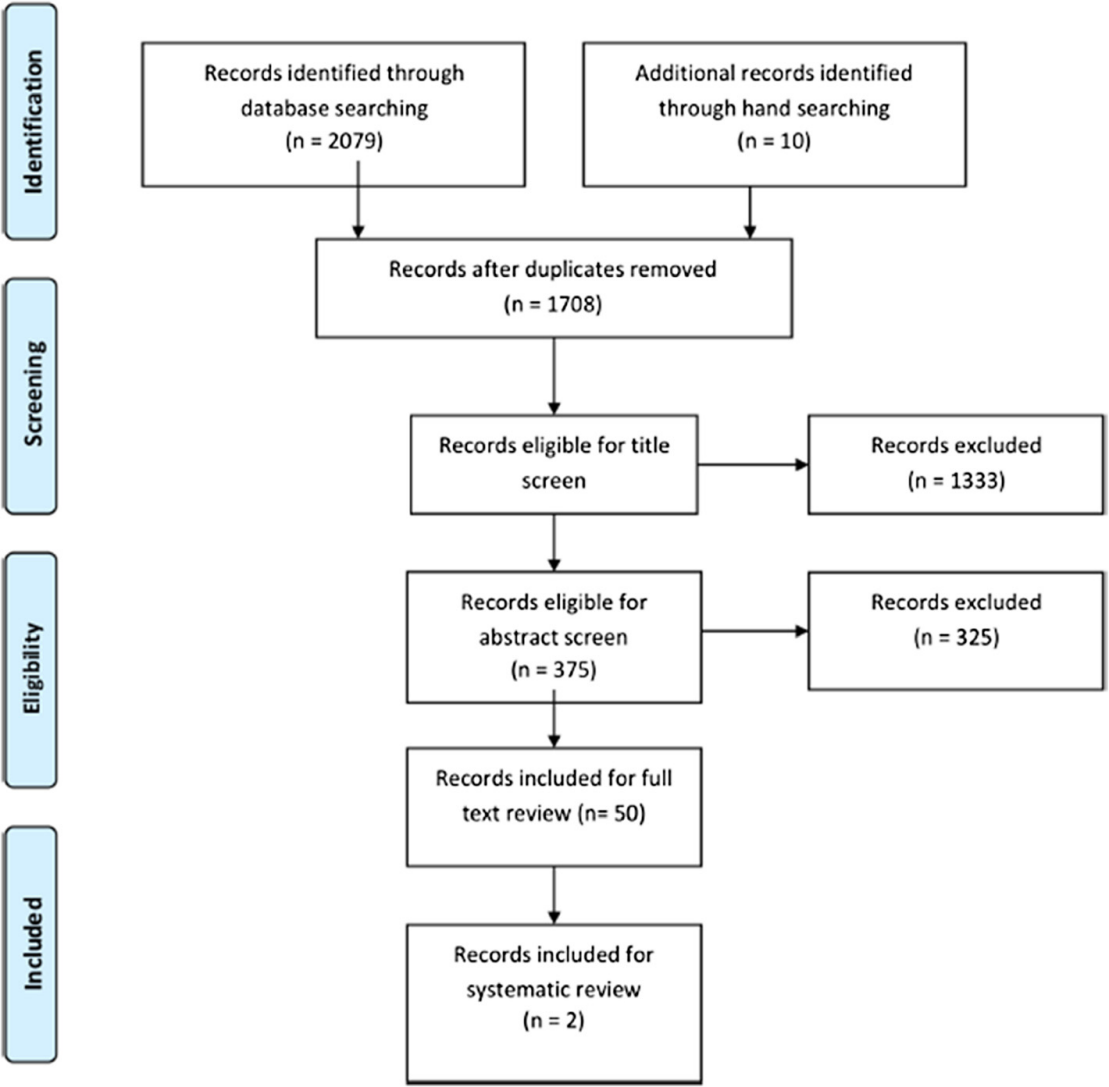
Search and screening process. PRISMA flow diagram

The search yielded 1708 articles eligible for screening. After the title screening, 375 papers were included for abstract screening. From this, 50 articles were identified as relevant for a full text review. Two of these met our inclusion criteria (Fig. 1).

### Study Designs and Intervention Descriptions

The two studies reported on educational interventions for healthcare providers aimed at increasing caregivers’ knowledge on FGM and related health consequences [11, 12]. Details of each of these studies are provided below. The intervention by Sangaré et al. (1998) was conducted in Mali, where FGM is prevalent. The objective of the intervention was to increase health workers’ knowledge about FGM and its health complications and to reduce the practice of FGM. A total of fourteen centers were selected—eight were the intervention centers and six were used as control sites. Fifty-nine providers (gynecologists, family planning providers, certified nurses, nurses in training, nurse aides, midwives, traditional birth attendants and health technicians) were interviewed before and after the education program. Forty-nine were interviewed without the training. 5390 women were observed during their consultations to collect information on the presence and type of FGM and related complications, and 1633 clients were interviewed following their consultations to evaluate the effect of any information, education and communication (IEC) activities on their attitudes and knowledge on FGM [11].

The second study (Jacoby and Smith, 2013) was undertaken with eleven certified nurse-midwives in Central Maine, in the United States. According to Population Reference Bureau’s data analysis of 2013, 507,000 women and girls have undergone or are at risk of FGM in the U.S. They come mainly from Egypt, Ethiopia and Somalia [13]. In Maine, 1603 migrant women have been estimated as having or being at risk of FGM [13]. The aim of the intervention was to increase certified nurse-midwives’ knowledge of the obstetric care of women and girls living with FGM [12]. Both studies are quantitative and evaluated the effectiveness of the intervention proposed by a survey before and after the educational program. Additional file 1: Table S1 provides an overview of the two studies.

### Educational intervention

In Mali, the study included a four-day educational program on FGM, and its health-related complications. The program included IEC activities, health talks at clinics, and visual aids for use during individual consultations with clients. Role playing was used to simulate counseling. The training was supervised by the organizations conducting the study [11].

The Maine intervention consisted of a PowerPoint presentation on the literature on FGM, case studies and information on the recommendations regarding the management of women with FGM of the American College of Obstetricians and Gynecologists. The program focused on FGM type III (infibulation), and included a roundtable discussion with a Somali cultural broker (an assistant with knowledge of Somali culture) and the members of the International Medicine Clinic of the Central Maine Medical Centre. A hands-on skills laboratory of defibulation was conducted. At the end, the participants received a laminated card on defibulation [12].

### Evaluation of the efficacy of the interventions

Both studies included interviews before and after the intervention [11, 12]. In Mali, evaluation was performed at baseline and end-line levels in both the intervention and control arms. No statistical analysis was performed. Health providers were interviewed with questionnaires assessing knowledge and attitudes concerning FGM. Socio-demographic profiles of the providers were also collected. One planned component of the evaluation was to observe the IEC activities during the consultations and interview clients after them. Although all the test sites were provided with flip charts on FGM designed for IEC activities, only two out of 14 sites conducted them. Only 4 % of the clients interviewed said they had received FGM-related information [11]. According to the authors, providers reported several reasons for not conducting the IEC activities, including the lack of an appropriate space, the lack of time, and discomfort in publicly broaching a taboo subject. The time span provided for IEC training was considered too short [11].

In the US study, the evaluation included surveys of the midwives before and after the training. The focus of the surveys was on the nine learning objectives of the course. The survey used a 5-point Likert scale to assess confidence in the following:

- Understand the historical, legal, ethical, and cultural significance of FGM and its prevalence worldwide.
- Recognize the four types of FGM and define their indicated respective management.
- Discuss the components necessary when counseling infibulated women about timing and necessity of defibulation and develop skills necessary for provision of culturally competent care.
- Document the type of FGM on the patient’s record correctly and plan for management, whether preconception, antepartum, or intrapartum.
- Comprehend basic principles for defibulation and repair.
- Define indications for intrapartum management of circumcised women.
- Discuss contraindications for a certified nurse-midwife to perform defibulation and repair.
- Display confidence in intrapartum defibulation and repair
- Discuss the role of the certified nurse-midwife in performing intrapartum defibulation and repair.

Percentages were used to assess the impact of the training in both studies [11, 12].

### Improved knowledge and attitude about FGM

The Malian study found that in the centers where training was offered, knowledge of FGM increased but this was not tested to determine statistical differences. The rate of caregivers unable to recognize FGM types dropped from 24 to 5 % and those who were able to list at least three complications of FGM increased from 50 to 72 %. Caregivers thinking that uncut women have “loose morals” decreased from 39 to 26 %; those presuming that men should marry a circumcised woman from 32 to 28 % and providers considering FGM a guarantee of virginity from 14 to 9 %.

Even though healthcare professionals in the control sites did not follow any training on FGM, also showed an improvement of knowledge. Those who were able to list at least three complications of FGM increased from 61 to 73 %. This is probably due to an increased sensitization to the subject due to the participation to the study and some information on FGM received during the first stage of the research. Some of the control health care professionals might have sought out some information on their own [11].

US midwives reported feeling more confident in the clinical and obstetric management of women with FGM. The level of self-confidence in the recognition of the four types of FGM and the management of each type improved from an average 2.36 to 4.18, with five being most confident. Their confidence in their ability to counsel women with type III went from 2.00 to 4.09. Their confidence in cultural competence increased from 2.36 to 4.09 and in performing defibulation from 1.54 to 3.54. Their ability to identify factors that are contraindications for defibulation went from 1.63 to 4.27 and to understand the historical, cultural, legal, and ethical considerations of FGM from 2.64 to 4.09 [12]. Again, no statistical tests for significance were performed.

### Attitude to FGM medicalization

The Malian study also evaluated healthcare providers’ attitude to medicalization of FGM. The final evaluation showed that caregivers (both in the intervention and control arms), less frequently thought that FGM carried out in a health facility is safe (from 35 to 17 %). 57 % thought medicalization should be forbidden, and over 20 % thought it should not be encouraged. After the intervention period about 10 % of providers believed performing FGM in a facility was “good practice”. and 30 % of the providers thought that the cutting of the clitoral hood is not risky. [11].

### Prevention of FGM

No information was collected on prevention capacities of healthcare providers having been trained.

## Discussion

### Main findings

Our systematic review resulted in two studies—and one was from the late 1990’s. Both studies evaluated outcomes regarding the improvement of healthcare professionals’ knowledge and attitude towards FGM and confidence in clinical management pre and post training. The Malian study also evaluated outcomes related to the medicalization of FGM. Neither evaluated the effects of the training on the quality of the care offered, the clinical outcomes of women attended, the satisfaction of the care received and prevention.

### Strengths and limitations

As far as we know, this is the first systematic review on interventions aimed at improving healthcare providers’ capacities of prevention and treatment of FGM. The strengths of our study are the inclusion of the grey literature, the retrieval of additional records through hand searching and by browsing webpages related to FGM and the absence of language restriction. The Malian study included in this systematic review was retrieved through the web-searches rather than standard peer-reviewed databases.

Our review was limited by the lack of available evidence. Despite placing no restriction on study design, only two studies met our inclusion criteria, and neither of these conducted statistical analysis to evaluate the outcomes. Because of that, the assessment of the quality of evidence was not the objective of our systematic review. The heterogeneity of study design, population included and outcome measures did not allow the computation of summary measures. As it is difficult to systematic search the grey literature, it is possible that we missed some interventions and evaluations.

### Interpretation

Evidence on effective and feasible interventions aimed at increasing providers’ capacities is extremely limited. A recent analysis of the evidence on knowledge, experiences and attitudes of health professionals toward FGM resulted in six areas for improvement for health care providers:

- Knowledge of FGM and its consequences;
- Adherence to FGM protocols and guidelines;
- Socially constructed acceptance of FGM;
- Knowledge of legislation and legal status of FGM;
- Condoning, sanctioning or supporting FGM; and
- Information and training to work with women and girls living with FGM.

These authors [14], in agreement with Dawson et al. [15], point to the key role of nurses and midwives in FGM management, and the need to strengthen evidence-based guidelines and professional individual and services health capacities.

Different e-learning tools for FGM have been implemented in high-income countries [6, 16]. However, their efficacy among caregivers has not been evaluated. FGM is a subject that requires specific cultural expertise to facilitate communication, counseling, care and prevention [7] and many healthcare professionals do not have any previous experience on the subject and will benefit from more training. Such novel on-line platforms provide broader training opportunities, but before they can be recommended, high quality evaluations are needed. The study of Jacoby and Smith showed that the majority of the trained midwives had no previous clinical experience of caring a woman with infibulation. The part of the program the participants found most powerful was the one with the Somali cultural broker [12]. In settings where FGM is not prevalent, but migrant women are living with FGM, collaborating with certified interpreters and cultural brokers could improve the training.

Iconographic material, including videos on defibulation [17], together with practical sessions of simulation and role play could improve ability, communication and confidence of caregivers [7].

In theory these interventions can work; however, evaluation will be needed. Evaluation measures should include knowledge and skills for providers working with women with FGM, as well as outcomes among women living with FGM, such as appropriate recording of FGM and type on clinical records, experiences of complications, surgical procedures linked to FGM, and obstetric and neonatal outcomes among others. Where hospital or national FGM registries and diagnostic codes are available, such as in United Kingdom [18], the information collected could be used in pre and post training evaluations.

In lower-income settings where most of the FGM is performed, providers can play an instrumental role in the perpetuation of FGM, as well as treatment and management of women living with FGM. In these settings, important measures may include health care workers’ attitudes towards FGM, reinfibulation and defibulation as well as their knowledge of FGM. Monitoring rates of refusal of defibulation and request of reinfibulation in case of FGM type III would also be important. So little research has been done in this area as it is probably difficult to monitor at long term healthcare workers clinical practice, clinical outcomes and satisfaction of clients on a subject like FGM, which is considered taboo among most of the communities and illegal in many countries.

Interventions aimed at sensitizing and training healthcare providers should be based on the assessment of the needs of the site of implementation. Healthcare providers that work with women with FGM in the diaspora or in countries where FGM is ritual can present similarities, such as the difficulty in recognizing and classifying FGM [19], but also differences and specific characteristics to be expressly addressed, such as the problem of medicalization in some African countries or the religious and cultural differences between Western caregivers and migrant patients. The study in Mali in fact, found that, before training, 35 % of the caregivers thought that FGM does not have health risks if performed in a safe environment [11]. Healthcare workers in low prevalence settings may be unfamiliar with cultural issues explaining the persistence of the practice or, could find it difficult or uncomfortable to ask about it because of the fear of embarrassing or causing distress to their patient [19].

## Conclusion

Approaches with healthcare workers in high and low prevalence settings need to consider prevention of FGM as well as treatment and care for women living with FGM.

With the paucity of evaluation data, it is evident that more research is needed to better understand the best way to effectively implement programs that train healthcare workers in best practices for women living with FGM.

## Additional file

Additional file 1: Interventions and outcomes of the two studies on interventions on FGM for healthcare professionals included in the systematic review. (DOCX 107 kb)

## Abbreviations

ACOG: American college of obstetricians and gynecologists
ASDAP: Association de soutien au développement des activités de population
CMMC: Central Maine Medical Center
DRSP: Direction régionale de santé publique
DSFC: Division de santé familiale et communautaire
FGM: Female genital mutilation
IEC: Information, education and communication
PRISMA: Preferred Reporting Items for Systematic reviews and meta-Analyses
TBA: Traditional birth attendants
WHO: World Health Organization
