# Supplementary material for: Interventions for healthcare providers to improve treatment and prevention of female genital mutilation: a systematic review
Source: BMC Health Serv Res. 2016 Aug 19;16:409. doi: 10.1186/s12913-016-1674-1 (PMC4992263; doi:10.1186/s12913-016-1674-1)
Supplement: Additional file 1: — Interventions and outcomes of the two studies on interventions on FGM for healthcare professionals included in the systematic review. (DOCX 107 kb) [file 12913_2016_1674_MOESM1_ESM.docx]

**Table 1. Interventions and outcomes of the two studies on interventions on FGM for healthcare professionals included in the systematic review. FGM= Female Genital Mutilation. IEC= Information, education and communication activities. TBA= Traditional birth attendants. ACOG= American College of Obstetricians and Gynecologists. CMMC=Central Maine Medical Center. DSFC= Division de santé familiale et communautaire. DRSP= Direction régionale de santé publique. ASDAP= Association de soutien au développement des activités de population.**

| **Author**  **Year**  **Country** | **Study design**  **Setting** | **Population**  **Sample size** | **Intervention** | **Comparison** | **Measures** | **Outcome reported** | **Strengths** | **Weaknesses** | **Quality** |
| --- | --- | --- | --- | --- | --- | --- | --- | --- | --- |
| Sangaré  1998  Mali | Case control.  Eight experimental health centers compared with 6 control health centers in Bamako and Bla (Ségou region).  Health centers include 1 hospital; urban, district and community health centers and referral centers | 108 health providers (OBGYN, family planning providers, certified nurses, nurses in training, nurses aides, midwives, TBA, health technicians): 59 received training, 49 no training. 1633 clients | 1) Four day training on female anatomy, FGM, related complications and treatments. IEC activities. Visual aids. Role playing to simulate counseling.  2) FGM-related IEC activities within health talks at the clinics and during individual consultations  3) Supervision by trained health staff of the organizations conducting the study* | No education program | Interviews pre and post training.  Observations of the IEC activities.  Interviews with clients after their consultations | Improved knowledge and attitude on FGM and consequences  Decreased attitude to FGM medicalization but in both experimental and control centers 30% think that FGM type Ia is not a health risk | Presence of a control group | Descriptive statistics only, no statistical analysis provided.  Limits of sampling: loss of follow up of 4 caregivers post training, low number of women exposed to IEC activities. Impact of training on clients could not be evaluated.  Control group not trained but sensitized to FGM in the first steps of the study. No information on differences of the two groups that could also affect knowledge and attitudes on FGM.  No randomization of caregivers assigned or not to education program | II-2 |
| **Author**  **Year**  **Country** | **Study design**  **Setting** | **Population**  **Sample size** | **Intervention** | **Comparison** | **Measures** | **Outcome reported** | **Strengths** | **Weaknesses** | **Quality** |
| Jacoby  2013  USA | Cohort study.  Hospital. Central Maine, US | Eleven midwives (50 midwives were invited to voluntarily take part to the training and study). | 1) Didactic information (Microsoft PowerPoint presentation on the literature on FGM, case studies, ACOG recommendations)  2) Cultural roundtable with a Somali cultural broker and members of the International Medicine Clinic of the CMMC  3) Hands-on skills laboratory  method of training on defibulation using pelvic models after reviewing the technique  4) Distribution of a laminated card on defibulation | No comparison | Survey pre and post training on 9 learning objectives of the course measured by a 5 point Likert scale going from no confident to very confident | Increased confidence on the clinical management of women with FGM, with a focus on FGM type III, defibulation and obstetric management | Well designed | Descriptive statistics only - no statistical analysis provided. | II-2 |
